# Preventing early childhood caries with silver diamine fluoride: study protocol for a randomised clinical trial

**DOI:** 10.1186/s13063-020-4088-7

**Published:** 2020-02-04

**Authors:** Sherry Shiqian Gao, Kitty Jieyi Chen, Duangporn Duangthip, May Chun Mei Wong, Edward Chin Man Lo, Chun Hung Chu

**Affiliations:** Faculty of Dentistry, The University of Hong Kong, Hong Kong, Hong Kong SAR China

**Keywords:** Silver diamine fluoride, Sodium fluoride, Children, Caries, Prevention

## Abstract

**Background:**

Silver diamine fluoride (SDF) solution is effective in arresting early childhood caries (ECC). Previous studies have suggested that it might exert a preventive effect in managing ECC. However, no well-designed clinical trials have yet been performed to study the effect of SDF on caries prevention. The objective of this randomised clinical trial is to determine whether 38% SDF solution is superior to 5% sodium fluoride (NaF) varnish in preventing new carious lesions in primary anterior teeth.

**Methods/design:**

This is a phase II, single-centre, randomised, double-blind, active-controlled, parallel-group pragmatic trial. The hypothesis tested is that 38% SDF would be more effective than 5% NaF in preventing new caries development in primary anterior teeth. Approximately 730 3-year-old children who are generally healthy and with parental consent will be recruited from Hong Kong kindergartens. This sample size will be sufficient for appropriate statistical analysis of a superiority trial with 90% power, allowing for a 20% drop-out rate. Stratified randomisation will be adopted for allocating the intervention. The intervention will either be 38% SDF or 5% NaF (as a positive control) therapy on primary upper anterior teeth. A single trained examiner will conduct a dental examination every 6 months until 30 months in kindergarten. Another operator will provide fluoride therapy immediately after each dental examination. The examiner, children and children’s parents will be blinded to the treatment allocation. A questionnaire survey will be conducted to study the children’s oral health-related behaviours and socioeconomic backgrounds. Chi-square tests, *t* tests, regression analyses and survival analyses will be adopted for data analysis.

**Discussion:**

The effectiveness of 38% SDF in preventing ECC remains uncertain. If the results are as anticipated, care standards using 5% NaF for ECC prevention will be changed. In addition, the results will be widely available and increase the adoption of SDF in other countries to reduce the global burden of ECC.

**Trial registration:**

ClinicalTrials.gov, NCT04075474. Registered on 30 Aug 2019.

## Background

Early childhood caries (ECC) is the single most common chronic childhood disease, and its development is a global health problem [1]. In the United States, nearly half of children have ECC before entering kindergarten [2]. More than half of 5-year-old Hong Kong children suffer from ECC, and around 90% of the caries are left untreated [3]. In the Philippines, the main reason for absence from school is dental caries [4]. Because the prevalence of dental caries in children aged 2 to 5 years has recently increased globally, the World Dental Federation (FDI) has prioritised this age group [5]. ECC causes pain and infection, and advanced caries will progress into the tooth pulp to eventually form a dental abscess [6]. Cases that remain untreated will lead to tooth loss, which can affect dentition. More importantly, poor dentition significantly affects children’s nutrition and consequently their growth, development and general health [7].

Although the FDI Oral Health Atlas has reported that millions of children are suffering from untreated ECC, along with a great shortage of community dentists, the present dental care delivery system cannot cope with the high ECC prevalence worldwide [8]. The FDI promotes the use of fluoride to prevent ECC in children through health-promotion strategies and programmes [9]. Although sodium fluoride (NaF) varnish is considered a standard of care in preventing ECC, a systematic review by the Cochrane Collaboration revealed that applying 5% NaF varnish (containing 22,600 ppm fluoride) is inadequate because it reduced only 37% of ECC development [10].

### Silver diamine fluoride solution

Silver diamine fluoride (SDF) is an ammonia solution containing fluoride and silver ions. Laboratory studies have found that 38% SDF can slow dentine and enamel demineralisation and inhibit the growth of common cariogenic bacteria [11, 12]. Moreover, it preserves collagen from degradation in demineralised dentin [13]. Based on these mechanisms, SDF has been used worldwide to arrest ECC. Systematic reviews of clinical studies have found that 38% SDF solution (containing 44,800 ppm fluoride) is effective in arresting ECC [14, 15]. In addition, clinical trials found that SDF exerted a preventive effect on the entire dentition when it was applied to only decayed anterior primary teeth [16], and it was effective for preventing caries in permanent teeth [17]. Therefore, apart from its caries-arresting effect, some researchers have also advocated for SDF therapy as an important prevention-centred caries-management strategy during critical early childhood periods. Nevertheless, a literature search in PubMed and ClinicalTrials.gov databases revealed that no well-designed clinical trials have studied the preventive effect of SDF against ECC.

### Objective

The objective of this randomised clinical trial is to assess the effectiveness of 38% SDF solution and determine if it is superior to 5% NaF varnish in preventing new carious lesions in the primary anterior teeth of young children.

### Hypothesis

The hypothesis under investigation is that the biannual application (every 6 months) topical application of a 38% SDF solution on the primary upper anterior teeth is superior to that of a 5% NaF varnish in reducing the number of sound tooth surfaces that become cavitated caries in kindergarten children at 30-month follow-up.

## Methods/design

### Trial design

**Fig. 1:**
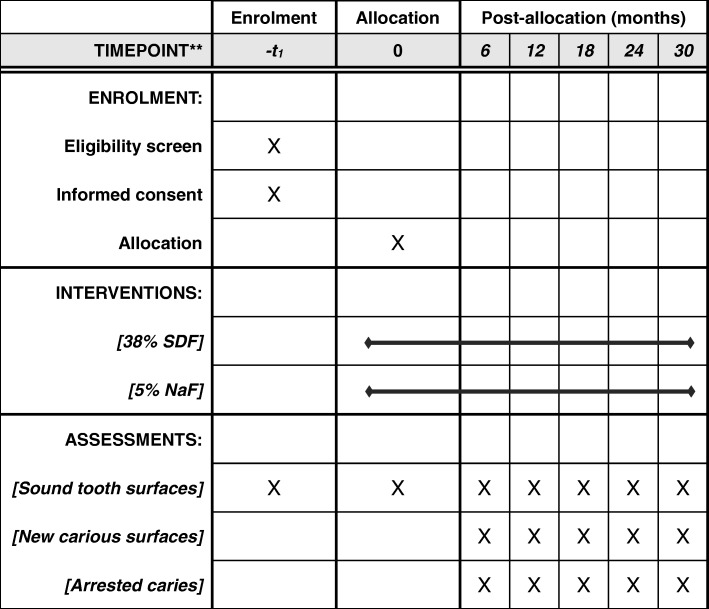
The schedule of enrolment, interventions, and assessments. NaF sodium fluoride, SDF silver diamine fluoride

This is a phase II, single-centre, randomised, double-blind, active-controlled, parallel-group pragmatic trial. The design and report of this clinical trial protocol follows the Standard Protocol Items: Recommendations for Interventional Trials (SPIRIT) statement (Additional file 1) [18]. The trial schedule is shown in Fig. 1.

### Setting

The Faculty of Dentistry of the University of Hong Kong has been providing outreach dental services to local kindergartens. Invitations will be sent to these kindergartens to explain the purposes and procedures of this study. After receiving confirmation of participation from a kindergarten’s headmaster, informative consent forms will be distributed to the parents of the eligible children (Additional file 2). Written parental consent will be collected before the dental examination and treatment take place.

### Participants

All of the children attending their first year of kindergarten from the participating kindergartens will be invited to join this study. The inclusion criteria will be children who: 1) are aged 3 to 4 years old; 2) are generally healthy; and 3) have parental consent. The exclusion criteria will be children who: 1) are uncooperative with the dental examination and treatment; 2) have major systemic diseases, such as porphyria; or 3) are on long-term medication such as antiepileptic drugs.

### Baseline and follow-up oral examinations

All of the baseline and follow-up oral examinations will be conducted in the kindergartens, primarily through careful visual inspection with the aid of a World Health Organization (WHO) CPI probe (405/WHO probe, Otto Leibinger, Mühlheim, Germany) and a disposable front-surface dental mirror with light-emitting diode intraoral illumination (MirrorLite, Kudos Crown Limited, Hong Kong, China).

The six upper anterior teeth will be the studied teeth. They will be cleaned and dried with a gauze sponge before evaluation of their caries status. Caries will be diagnosed at the cavitation level. A surface without any cavitated lesions will be considered sound. The carious lesion will be gently explored with a CPI probe in the lesion’s centre. Great care will be taken to avoid tooth damage during the probing. A carious lesion will be recorded as active if softness is detected upon gentle probing. If the lesion is hard when probing, it will be classified as an arrested caries [16, 17]. The surface of each upper anterior tooth will be recorded as sound or carious (active or arrested). Apart from the studied teeth, the decayed, missing (due to caries) and filled primary tooth (dmft) index will be used to record each child’s caries experience (tooth level). Other tooth statuses, such as tooth discolouration and hypermobility, of all primary teeth will be recorded as well. Oral hygiene status will be measured using the visible plaque index [19]. The buccal and lingual surfaces of six index teeth (55, 51, 63, 71, 75 and 83) will be examined. The same examiner will conduct the follow-up oral examinations every 6 months for 30 months in the kindergartens using the same equipment, procedure and diagnostic criteria as those used in the baseline examination. The intraexaminer agreement on the caries and plaque assessment will be monitored and tested in 10% of the children at each stage of the study.

### Intervention

The children will receive either 38% SDF solution or 5% NaF varnish treatment on all of their upper anterior tooth surfaces. An independent operator will use a microbrush (MICROBRUSH, Grafton, WI, USA) to apply the SDF solution (Advantage Arrest, Elevate Oral Care, FL, USA) or NaF varnish (Duraphat Varnish, Colgate-Palmolive, NY, USA) according to the assigned treatment group. The SDF solution or NaF varnish will be applied once every 6 months for 30 months right after the oral examination. The kindergarten teacher will be instructed not to allow children in both groups to eat or drink for half an hour after the fluoride application. There will be no special criteria for discontinuing or modifying allocated interventions. Because the intervention is administered by a dentist, there are no strategies to improve adherence and monitoring of adherence. Implementing fluoride treatments will not require alteration to usual care pathways (including use of any medication) and these will continue for both trial arms.

### Randomisation, treatment allocation and allocation concealment

The participating kindergarten children will first be categorised as either: 1) having an increased caries risk, which is defined as having caries experience (dmft >0); or 2) having a low caries risk with no caries experience (dmft = 0). The children will then be allocated by a stratified randomisation method at the subject level with two strata (increased and low caries risk) using a personal computer into the following two groups in blocks of eight children for fluoride treatment every 6 months: group SDF (38% SDF solution) and group NaF (5% NaF varnish). There will be no negative control group for ethical reasons. A statistician will keep the random number sequence, and opaque sealed envelopes will be used to conceal the allocation sequence until the interventions are assigned.

### Blinding

In this randomised clinical trial, the examiner, the children and their parents will not be informed about the treatment group allocation throughout the study. Each assigned fluoride treatment will be applied after the oral examination by an independent operator. If parents request the treatment history of their child, unblinding is permissible. An unblinded research assistant will disclose treatment allocation to the parents, and the child will be excluded from the study.

### Outcome measure

The primary outcome measure will be sound surfaces of the six anterior teeth at baseline that develop or do not develop caries at the 30-month examination. The secondary outcomes measured will be the number of carious surfaces developed during the study that become arrested, caries experience (dmft index), increment in the number of nonvital teeth and number of hypermobile teeth at 30-month follow-up.

### Effect modification

A parental questionnaire that has been used in previous studies [3, 20] will be administered at baseline and again at the 30-month follow-up visit regarding their children’s oral hygiene habits (e.g. toothbrushing), fluoride agent use (e.g. fluoride toothpaste), diet habits (e.g. bottle feeding), snacking habits, dental visit behaviour, parental educational level, family income and family status (single- or both-parent households).

### Sample size calculation

The mean number of cavitated teeth of a 3-year-old child was five in our previous study [16]. The mean number of new cavitated caries surfaces found in the 62 children in the SDF group was 0.47 (standard deviation (SD) 0.87), and the mean new cavitated caries surfaces in the 61 children in the NaF group was 0.70 (SD 0.84) at the 30-month review. This difference corresponded to a prevented fraction of 33% more in the SDF group when compared to the NaF group, which is considered clinically significant. With the statistical power set at 0.9 and type I error rate of 5% for a two-sided test, the required sample size would be 292 for each group. Considering a dropout rate of 20%, the total number of children to be recruited at baseline should be 365 in each group. Therefore, a total of 730 children need to be recruited at baseline examination for this trial.

### Data management

Two people will independently enter the collected data into an Excel file (by double entry) and the data will be compared to minimise data entry errors. A statistician will oversee the data entry, data checking and analysis for this project. The intraexaminer agreement in caries diagnoses at each time point will be assessed using Cohen’s Kappa statistics. This trial will employ an intention-to-treat analysis. A per-protocol analysis will be performed if the number of children who stray from the protocol (for instance, by not adhering to the prescribed 6-month intervention, or by being withdrawn from active treatment) differs between the two groups. In that case, only the patients who complete the entire clinical trial according to the protocol will be counted towards the final results. Sensitivity analysis will also be considered by means of multiple imputations for missing values. The level of statistical significance for all two-sided tests will be set at 0.05. For one-sided tests, the level of statistical significance will be set at 0.025.

### Data analysis

The statistical software SAS for Windows (SAS Institute Inc., USA) and SPSS for Windows (IBM Corporation, USA) will be used for the data analyses. Statistical analysis will be performed at the subject and tooth surface levels. An interim analysis will be performed at 18-month follow-up.

#### Subject-level analysis

Although the data for the primary outcome may not be normally distributed [16], the distribution of the mean would be normal according to the central limit theorem with a relatively large sample size. At the subject level, the outcome includes the percentage of children with no new caries developed over the study period as well as the mean number of new caries. Chi-square tests will be used to test the between-group differences in the proportion of subjects with new caries development and in the proportion of surfaces with new cavitations; *t* tests will be used to study the between-group differences in the mean number of new caries and in the increases in the numbers of nonvital teeth and of hypermobile teeth across the follow-up examinations. This research will also study if the treatment effects differ by patient characteristics (effect modification); the variables that may modify the treatment effects on the outcome variable include sex, baseline caries experience, treatment group assignment, oral hygiene habits, fluoride agent use, diet habits, snacking habits, dental visit behaviour, parental educational level, family income and family status (single- or both-parent households). As the outcome variable may not be normally distributed, Poisson or negative binomial regression will be considered to study the effect’s modification [21]. Aside from the primary outcome, *t* tests will also be performed to study the treatment’s effects on the number of arrested carious surfaces at the 30-month examination between the two groups.

#### Surface-level analysis

To compare the differences in the time to develop cavitated carious lesions at the tooth surface level between the two treatment groups, a multilevel survival analysis will be adopted for the interval-censored data (because the time to develop the cavities cannot be observed exactly but falls within the interval between the two examinations). This analysis will account for the possible correlation (clustering) between the observations of multiple surfaces from the same child.

### Ethical consideration

Ethical approval has been sought from the Institutional Review Board of the University of Hong Kong/Hospital Authority Hong Kong West Cluster (HKU/HAHKW IRB) (UW18–619). Written consent will be obtained from the parents of each participating child. All of the participants will have the right to withdraw from the study at any time by informing the primary investigator. Withdrawal from the study will not influence the participants’ right to receive other services, such as oral health education. In general, the study will pose minimal risk to the participating children. Professional training will be provided to the field workers in order to minimise the risk. If one life-threatening case has been identified or more than 30% of the participants have severe systemic side effects, the trial will be stopped. All the personal information about potential and enrolled participants will be kept confidential in a personal computer. Only the investigators will have the right to access the dataset.

## Discussion

This is a phase II, single-centre, randomised, double-blind, active-controlled, parallel-group pragmatic trial that will assess the effectiveness of 38% SDF in preventing new carious lesions in primary anterior teeth. Because 5% NaF varnish is considered a standard of care for preventing ECC, we will use 5% NaF as a positive control group to assess whether 38% SDF is superior to this standard of care. There will be no negative control group in this study because every enrolled child should have the right to receive an effective preventative strategy. Since 38% SDF has a significantly higher fluoride concentration (44,800 ppm) than 5% NaF (22,600 ppm), our hypothesis is that 38% SDF will have a superior effect over 5% NaF in preventing ECC. If the results are as anticipated, this will help to change the standard of care. In addition, the results will be widely available and increase the adoption of SDF in other countries to reduce the global burden of ECC.

The latest survey found that ECC affected 55% of 5-year-old Hong Kong kindergarten children [3]. The ECC prevalence among 3-year-old kindergarten children was 22% [22]. A significant increase in ECC prevalence can be identified during the kindergarten life of Hong Kong children. Notably, 70% of the 5-year-old children suffering from ECC had their caries develop on their upper anterior teeth [3]. Therefore, we decided to provide a pragmatic intervention to prevent ECC progression by applying fluoride to children when they enter kindergarten (i.e. at 3 years old) and in the sites with high risk (i.e. upper anterior teeth).

This trial will be an outreach-based study. All of the clinical procedures will be performed in kindergartens. Therefore, the use of dental radiographs to detect dental caries is unavailable and unsafe under this setting. The examiner will use visual–tactile inspection without x-rays to assess the presence of carious lesions at baseline and the follow-up examinations, which other clinical caries studies have demonstrated to be practical and reliable [16, 20].

In this study, we assess the preventive treatment (38% SDF or 5% NaF) as the primary assessed independent variable regarding children’s ECC development. Apart from that, other clinical parameters, such as children’s caries experience (dmft index), will also be included as an effect modifier. Studies have reported that children’s oral health-related behaviours (toothbrushing frequency, use of fluoride products and bottle-feeding behaviours) and their socioeconomic status (family income and parental education level) are risk factors for the presence of ECC [3, 22]. Therefore, all of this information will be collected through a parental questionnaire survey, and those factors will be included in a regression analysis to see if they are related to the primary outcome (caries prevention effect).

We will use visual inspection to diagnose dental caries, rather than taking radiographs. Moreover, both SDF solution and NaF varnish have been approved as safe for clinical use, and the procedure of applying these treatments is noninvasive. Therefore, limited ethical concerns have been raised in this study, although the participants will be recruited at a young age. The participating children will enter primary school after this trial. They will receive dental care from the Department of Health through the School Dental Care Service.

## Trial status

This clinical trial was registered in ClinicalTrials.gov under the registration number NCT04075474 in September 2019. This protocol is version 2 developed on 14 January 2020. The recruitment for participation started on 18 September 2019. The anticipated date of recruitment completion is 31 January 2020.

## Supplementary information

**Additional file 1:** Standard Protocol Items: Recommendations for Interventional Trials (SPIRIT) 2013 checklist.

**Additional file 2:** Parental consent form.

## Data Availability

The data sets generated and/or analysed during the current study are available from the corresponding author on reasonable request. The results of each follow-up examination will be shared to each participant by a report to the parents. The interim and final results will be shared to other researchers by publications and presentations in international conferences.
