# Supplementary material for: Preventing early childhood caries with silver diamine fluoride: study protocol for a randomised clinical trial
Source: Trials. 2020 Feb 4;21:140. doi: 10.1186/s13063-020-4088-7 (PMC7001262; doi:10.1186/s13063-020-4088-7)
Supplement: Supplementary file 2 — Additional file 2: Parental consent form. [file 13063_2020_4088_MOESM2_ESM.docx]

1^st^ September 2019

Dear Parents of preschool children,

**Preventing tooth decay in preschool children**

The Faculty of Dentistry of The University of Hong Kong (HKU) is conducting a caries control research on preschool children. The aims are to prevent the formation of dental caries (tooth decay) by silver diamine fluoride (SDF) or sodium fluoride (NaF) and to see which shows a better effect in preventing caries in children. Your child’s kindergarten is one of the participating kindergartens, and we will provide participating children a 30-month caries preventive and arresting treatment.

The details of the activity would be as follows:

- Target: K1 students;
- Proposed period: Semester year 2019-2022 including routine oral examination and fluoride treatment at six-month intervals
- Activities: (1) oral health education; (2) parental questionnaire survey; (3) oral examination; (4) SDF or NaF treatment.

These activities will be carried out in your child’s kindergarten. Each child will be randomly allocated to a treatment group of SDF or NaF treatment. The oral examination and treatment will take 5 minutes. No radiographs will be taken. No lifestyle or dietary restriction is required during the study period. Each participating child will receive a report regarding his/her oral health status. Subsequent semi-annual oral examination will be performed for the participants. Those who need any other dental services such as extraction can be treated by their own dentist at their own cost.

This study will provide fluoride treatment to all upper front teeth of your child (6 teeth in total), in regardless of the presence of tooth decay. If there is tooth decay presented, the decayed lesion may become black after the fluoride treatment. The black stain will not fade away until the replacement of milk teeth. However, please be noted that the black stain means the tooth decay has been successfully stopped from progressing. If your child does not have tooth decay, the teeth will not turn black after the fluoride treatment.

It is up to you to decide whether or not to let your child to take part. If you decide to take part in the study, please fill in the questionnaire attached as well as authorise us to provide a dental examination and fluoride treatment to your children in school time. You are still free to withdraw at any time and without giving a reason. This will not affect the standard of care you receive in the future.

You have the rights of access to personal data and publicly available study results, if and when needed. Under the laws of Hong Kong (in particular the Personal Data (Privacy) Ordinance, Cap 486), you enjoy or may enjoy rights for the protection of the confidentiality of your personal data, such as those regarding the collection, custody, retention, management, control, use (including analysis or comparison), transfer in or out of Hong Kong, non-disclosure, erasure and/or in any way dealing with or disposing of any of your personal data in or for this study. For any query, you should consult the Privacy Commissioner for Personal Data or his office (tel no. 2827 2827) as to the proper monitoring or supervision of your personal data protection so that your full awareness and understanding of the significance of compliance with the law governing privacy data is assured.

By consenting to participate in this study, you expressly authorise:

1. the principal investigator and his research team and the ethics committee (Institutional Review Board of the University of Hong Kong / Hospital Authority Hong Kong West Cluster) responsible for overseeing this study to get access to, to use, and to retain your personal data for the purposes and in the manner described in this informed consent process; and
2. the relevant government agencies (e.g. the Hong Kong Department of Health) to get access to your personal data for the purposes of checking and verifying the integrity of study data and assessing compliance with the study protocol and relevant requirements.

It is possible that when taking part in the oral examination, your child may feel discomfort. If your child is too unwilling to be checked, the oral examination will stop immediately. If your child is harmed by taking part in this study, there are no special compensation arrangements. If your child is harmed due to someone’s negligence, then you may have grounds for a legal action. Regardless of this, if you wish to complain about any aspect of the way your child has been approached during the course of this study, the normal health service complaints mechanisms may be available to you.

After examination, each of the participants will receive a set of souvenir. We hope that the oral examination and fluoride treatment will help you to understand the oral health status of your child. This study has been reviewed by Institutional Review Board of the University of Hong Kong / Hospital Authority Hong Kong West Cluster. If you have any enquiry, please contact me at 2859 0287 during office hours. Please complete the following consent if you agree to participate in this study. Thank you very much.

This research project will be provided for free by the Faculty of Dentistry, HKU, with no commercial interest. We sincerely ask for your approval to allow your child to participate. You are entitled to refuse to participate in this research project and this will not affect your child’s rights of medical and dental services. You can also withdraw from the research any time. All information and data collected will be kept confidential and use for research purpose only. If you have any questions, you are welcome to reach Prof. C.H. Chu by phone 28590287 or through e-mail chchu@hku.hk.

Thank you.


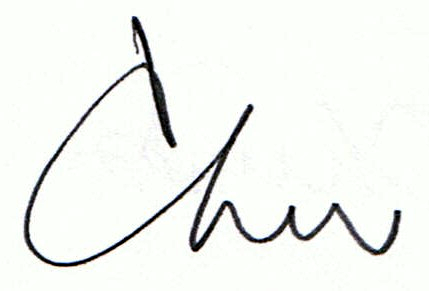


Prof. C.H. Chu, Clinical Professor,

Faculty of Dentistry, The University of Hong Kong

**PATIENT/SUBJECT CONSENT FORM**

| Child name: ____________________ | | | | | | |
| --- | --- | --- | --- | --- | --- | --- |
| Title of Project: **Preventing early childhood caries with silver diamine fluoride**  Name of Researcher: Prof. Chu Chun Hung | | | | | | |
|  | | | | *Please tick ✓ in the box* | | |
| 1. I confirm that I have read and understood the information sheet for the above study and have had the opportunity to ask questions. | | | |  | | |
| 1. I understand that my participation is voluntary and that I am free to withdraw any time, without giving any reason, without my medical care or legal rights being affected. | | | |  | | |
| 1. I understand that sections of any of my medical notes may be looked at by responsible individuals from Faculty of Dentistry, The University of Hong Kong or from Institutional Review Board of the University of Hong Kong / Hospital Authority Hong Kong West Cluster.   I give permission for these individuals to have access to my records. | | | |  | | |
| 1. Regarding this three-year study: | | | |  | | |
| - 1. I agree to let my child undergo oral examination and fluoride treatment; | | | |  | | |
| - 1. I agree to complete the questionnaire; | | | |  | | |
| - 1. I agree to have photos taken of the teeth of my child (not including face). | | | |  | | |
|  |  |  |  |  |  |  |
| Name of Parent / Guardian | | | Date |  | Signature |  |
|  | Prof. Chun Hung Chu |  | September 2019 |  | 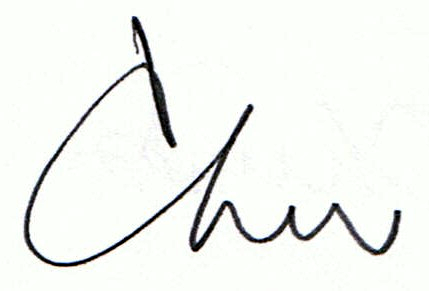 |  |
|  | Researcher |  | Date |  | Signature |  |
|  |  |  |  |  |  |  |

**幼童蛀牙防治研究**

尊敬的家長：

香港大學牙醫學院現在始進行一項幼童蛀牙防治研究。 貴子女的幼稚園是這項研究其中一間參與的幼稚園，本研究目標是以38%氟化氨銀或5%氟化鈉預防幼童蛀牙形成，並比較此兩種藥物的療效，讓牙科醫生可以根據此研究決定何種治療方式能更好減少幼兒蛀牙的形成。

本研究活動內容包括：

- 研究對象：幼稚園K1學生；
- 研究時間：2019-2022年度為幼兒提供每六個月一次的口腔檢查以及氟素治療；
- 研究活動：（一）口腔健康教育及口腔檢查、（二）家長問卷調查、（三）氟化氨銀治療，或氟化鈉治療、（四）提供免費牙刷和含氟牙膏以提高口腔護理意識。

研究活動將於幼稚園內進行。口腔檢查將需時5分鐘。檢查情況會被記錄。過程中將不進行放射性檢查； 貴子女亦無須改變原有之生活習慣或飲食習慣。檢查後，閣下將收到 貴子女的口腔報告。若 貴子女有其他牙科服務需要，例如拔牙，家長可以帶其子女接受自費牙科治療。

此項目會為貴子女的所有上門牙（共計6隻）進行氟素治療（無論有無蛀牙存在）。如貴子女已有蛀牙存在，經過治療的牙齒有機會變黑，且黑色不會褪去，直至更換恆齒。但請留意，蛀牙變黑則代表它已經停止發展，是一個治療成功的表現。如貴子女沒有蛀牙，接受藥水治療後，牙齒並不會變黑色。

閣下可以決定同意或不同意 貴子女參與此項研究。如果同意參與研究，需要閣下花費數分鐘時間填寫問卷及簽署同意書。閣下可在任何時間在不提供原因的情況下選擇退出此項研究，閣下於是次研究作出的任何決定不會影響貴子女接受牙科服務的權利。

有需要的話，每個研究參與者都有權利獲得其個人的數據以及公開報告的研究結果。根據香港法律（特別是「個人資料（私隱）條例」，第486章），您有權對您個人資料進行保密，如在本項研究中或與本項研究有關的個人資料的收集、保管、保留、管理、控制、使用（分析或比較）、在香港內外轉讓、不披露、消除和/或任何方式處理。如有任何問題，您可以諮詢個人資料私隱專員或致電到其辦公室（電話號碼：2827 2827），以適當監管或監督您個人資料保護，以便您能完全認識和瞭解確保遵守法律保護隱私資料的意義。

同意參與該項研究，您明確作出以下授權:

1. 為了監督該項研究，授權主要研究者及其研究團隊和倫理委員(香港大學及醫管局港島西醫院聯網研究倫理委員會)根據本項研究和本知情同意書規定的方式獲得、使用並保留您的個人資料；並且
2. 為了檢查和核實研究資料的完整性、評估研究協定與相關要求的一致性，授權相關的政府機構（如香港衛生署）可獲得您個人資料。

在檢查過程中，貴子女可能會感到不適。如 貴子女非常不願接受檢查，口腔檢查將會馬上停止。如 貴子女在參與此項研究過程中受到傷害，不會有其他額外賠償。如 貴子女是因研究員的疏忽大意而受到傷害，閣下可以採取法律措施。除此之外，如果閣下希望對研究過程中的任一方面進行投訴，可向一般健康服務投訴機制諮詢。

完成檢查後， 貴子女將會獲得一份小禮品。此項研究經由香港大學及醫管局港島西醫院聯網研究倫理委員會批准。閣下如有任何疑問，請在辦公時間內致電2859 0287朱振雄醫生。如閣下決定參與此項研究，請簽署知情同意書並交回。謝謝。

本研究計劃由香港大學牙醫學院免費提供，並沒有任何商業利益。我們現在誠邀閣下允許 貴子女參加。這項研究純為自願性質。本研究所收集的資料將會保密及只供研究使用。並們會通家長帶他們子女到任何牙科診所接受自費治療。如有任何問題，歡迎您致電 2859 0287 或通過電郵 chchu@hku.hk 與朱振雄牙科醫生聯系，多謝。


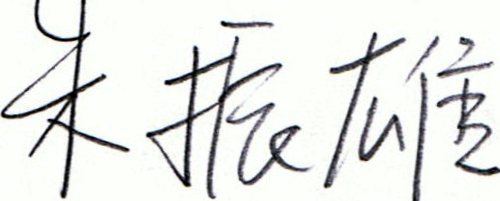


朱振雄牙科醫生

香港大學牙醫學院臨床教授

二零一九年九月一日

**家長/ 監護人同意書**

| 貴子女姓名： _________________  研究項目名稱： **幼童蛀牙防治研究**  研究者： 朱振雄醫生 | | | | | | | | |
| --- | --- | --- | --- | --- | --- | --- | --- | --- |
|  | | | | | | | 請在方格中打剔 *✓* | |
| 1. 本人已閱讀並理解有關以上研究的知情書，並有權利對其提出疑問。 | | | | | | |  | |
| 1. 本人明白本人的參與基於自願原則，並且有權利隨時在不提供原因的情況下退出研究，而本人接受健康服務的權利及法定權利將不受影響。 | | | | | | |  | |
| 1. 本人明白本人研究中的任何一部分健康紀錄將會被香港大學牙醫學院或香港大學及醫管局港島西醫院聯網研究倫理委員會之研究人員查看。 本人允許以上人員查看本人子女的紀錄。 | | | | | | |  | |
| 1. 關於此項為期三年的研究： | | | | | | |  | |
| - 1. 本人同意讓我的孩子接受口腔檢查及氟素治療，檢查情況將會被記錄。 | | | | | | |  | |
| - 1. 本人同意完成問卷調查。 | | | | | | |  | |
| - 1. 本人同意為我的孩子的牙齒影相（不包括面部）。 | | | | | | |  | |
|  |  | |  |  |  |  | |  |
|  | 監護人姓名（正楷） | |  | 日期 |  | 簽名 | |  |
|  | 朱振雄牙科醫生 | |  | 2019年9月 |  | 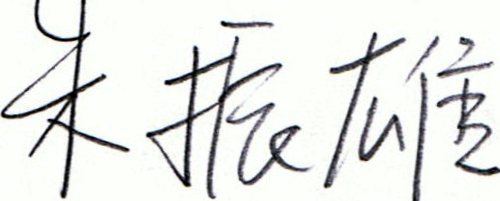 | |  |
|  | 研究者 |  | | 日期 |  | 簽名 | |  |
|  | | | | | | | | |
